# Digital solutions for mental health care during the COVID-19 pandemic: A systematic qualitative review and swot analysis

**DOI:** 10.1192/j.eurpsy.2021.686

**Published:** 2021-08-13

**Authors:** I. Sarajlic Vukovic, G. Valeriani, G. Mijaljica, A. Osmanovic Thunström, A. Gonzales

**Affiliations:** 1 Affektive Psychiatri, Sahlgrenska University Hospital, Gothenburg, Sweden; 2 Refugee Health Center, Ostergotland Region, Norköpping, Sweden; 3 Crisis And Trauma Service, VGR, Gothenburg, Sweden

**Keywords:** telehealth, COVID-19, healt- equity, review

## Abstract

**Introduction:**

Since its early stages, the Covid-19 outbreak has posed immense challenges for effective, scalable and rapid interventions. Telehealth approaches have been considered as key part of an effective pandemic response.

**Objectives:**

The aim of this systematic review was to evaluate the role of digital solutions in fighting the mental health needs during COVID-19 outbreak.

**Methods:**

This review was conducted through searching four databases including PubMed, Scopus, Web of Science, and Science Direct. Inclusion criteria included studies clearly defining any use of telehealth services in all aspects of mental health care during COVID-19 outbreak, published from December 31, 2019 to October 31, 2020, written in English language and published in peer-reviewed journals. Narrative synthesis was undertaken to summarize the findings according a SWOT (strengths, weaknesses, opportunities, threats) analysis.

**Results:**

62 studies met the inclusion out of the 278 search results. Data converged on: strengths in minimizing the risk of Covid-19 transmission, reduction of travel time and costs, comparable effectiveness to in-person care; weaknesses i.e. decreased ability to detect non-verbal cues, lower therapeutic alliance, possible technical connection problems; opportunities in improving the healthcare system and expanding its accessibility for patients also for the future; threats such as privacy and legal issues, and risk to overlook vulnerable populations (e.g. elderly, marginalized ethnic minorities).

**Conclusions:**

In the midst of a global mental health emergency, telehealth may represent a “virtually perfect” solution. However, further implementations facing issues of quality, justice and healthcare equity are required to ensure that all patients receive the care they need.

